# Editorial for the Special Issue on Smart Devices and Systems for Vibration Sensing and Energy Harvesting

**DOI:** 10.3390/mi14010173

**Published:** 2023-01-10

**Authors:** Kai Tao, Yunjia Li

**Affiliations:** 1 Ministry of Education Key Laboratory of Micro and Nano Systems for Aerospace, Northwestern Polytechnical University, Xi’an 710072, China; 2 Research and Development Institute in Shenzhen, Northwestern Polytechnical University, Shenzhen 518057, China; 3 School of Electrical Engineering, Xi’an Jiaotong University, Xi’an 710049, China

The Internet of things (IoT) poses new challenges for sensors and their power systems [1,2]. The deployment of large numbers of sensor nodes requires the sensor to work for a sufficient period without battery replacement. As self-sustained power sources, micro/nano energy harvesting systems can capture and transform unused ambient energy into electrical energy [3,4,5]. They have been regarded as an alternative to conventional electrochemical batteries, which will pave the way for actualizing energy-autonomous devices and intelligent monitoring activities. Integrating micro/nano power sources with IoT will be a revolutionary technology in the following decades. In addition, as many of the vibration energy harvester devices are sensitive to vibration, they are inherently considered as excellent candidates for vibration sensing.

This Special Issue focuses on state-of-the-art vibration sensing and energy harvesting technologies. After being carefully reviewed, 11 articles have been accepted for publication in this Special Issue [6,7,8,9,10,11,12,13,14,15,16] that can be divided into three aspects: (i) new materials for vibration energy harvesting applications, (ii) design and fabrication of vibration energy harvesters (VEHs), and (iii) system-level integration and testing of VEHs. 

New materials are crucial for breaking the bottleneck in the performance of the VEHs. Zhang et al. investigated the influence of ferromagnetic material on the output characteristics of the Halbach array energy-harvesting structure. After adding the iron sheet, the energy-harvesting efficiency of the Halbach array can be significantly improved [6]. Chen et al. proposed a novel double-network ionic hydrogel and fluorinated ethylene propylene (FEP) electret-based tactile sensor. Combining with a corona-charged FEP film, the output performance of the sensor is significantly boosted by 156.3% through the hybrid of triboelectric and electrostatic effects [7].

Structural design is another essential aspect for boosting the output performance of the VEHs. A torsional oscillating magnet-based vibration energy harvester was reported by Wang et al. Microfabricated silicon torsional springs contributed to the torsional movement of the magnet, which effectively reduced the footprint of the device and achieved an output power of 6.9 μW with a size of 1 cm × 1 cm × 1.08 cm [8]. Li et al. proposed an electromagnetic vibration energy harvester with tunable resonance frequency based on the stress modulation of flexible springs. By physically pulling and pushing the springs via a pair of metallic fixtures, the total tuning frequency range is 56 Hz (74–130 Hz) [9]. To improve the energy harvesting performance of an energy harvester, Chen et al. proposed a novel bistable piezoelectric energy harvester by introducing a linear-arch beam, the dynamics model of the system was established, and the simulation was conducted to verify the feasibility [10]. Furthermore, Zhang et al. reported a tri-stable piezoelectric energy harvester based on a linear-arch composite beam. The experiment result shows that the system can be mono-stable, bi-stable, and tri-stable by adjusting the horizontal or vertical spacing of the magnets [11]. Xu et al. utilized the vibration energy to drive a single-phase ultrasonic motor directly. The motor obtains advantages in structure miniaturization and circuit simplification [12]. Dong et al. reported stretchable strain sensors with a controllable negative resistance sensitivity coefficient by designing a silica gel/CNTs/silica gel sandwich structure [13]. 

Optimization of the system-level integration methodologies of VEHs is important for their practical applications. Cao et al. proposed an electrostatic‒piezoelectric‒electromagnetic hybrid vibrational power generator. By designing the energy management circuit at the rear end of the device, the packaged device can directly export a 3.3 VDC voltage to supply power to most of the sensing equipment [14]. A novel hydraulic interconnected regenerative suspension was reported by Guo et al. [15]. The root means square values of the bounce and roll acceleration of the proposed system are 64.62% and 11.21% lower than that of a standard suspension, respectively. The suspension system could output 186.93, 417.40, and 655.90 W at speeds of 36, 72, and 108 km/h for an off-road vehicle on a Class-C road, respectively. Apart from the design and fabrication processes, the reliability study on the VEHs is also necessary for the application of vibration energy harvesters. For this purpose, Li et al. presented a micro-tester for measuring the reliability of the energy harvester and other MEMS devices in controlled environmental conditions [16]. The system could generate a temperature range of 0–120 °C and a humidity range of 20–90% RH (0–55 °C) within a small footprint and weight.

In conclusion, the researchers reported large numbers of innovative research progresses in materials, device configuration, and system-level VEHs. This Special Issue gathers the latest developments in smart devices and systems for vibration sensing and energy harvesting applications, which could serve as a good reference for VEH research.
